# Glutamine metabolism regulates autophagy-dependent mTORC1 reactivation during amino acid starvation

**DOI:** 10.1038/s41467-017-00369-y

**Published:** 2017-08-24

**Authors:** Hayden Weng Siong Tan, Arthur Yi Loong Sim, Yun Chau Long

**Affiliations:** Department of Biochemistry, Yong Loo Lin School of Medicine, National University of Singapore, Singapore, Singapore

## Abstract

Activation of autophagy and elevation of glutamine synthesis represent key adaptations to maintain amino acid balance during starvation. In this study, we investigate the role of autophagy and glutamine on the regulation of mTORC1, a critical kinase that regulates cell growth and proliferation. We report that supplementation of glutamine alone is sufficient to restore mTORC1 activity during prolonged amino acid starvation. Inhibition of autophagy abolishes the restorative effect of glutamine, suggesting that reactivation of mTORC1 is autophagy-dependent. Inhibition of glutaminolysis or transamination impairs glutamine-mediated mTORC1 reactivation, suggesting glutamine reactivates mTORC1 specifically through its conversion to glutamate and restoration of non-essential amino acid pool. Despite a persistent drop in essential amino acid pool during amino acid starvation, crosstalk between glutamine and autophagy is sufficient to restore insulin sensitivity of mTORC1. Thus, glutamine metabolism and autophagy constitute a specific metabolic program which restores mTORC1 activity during amino acid starvation.

## Abstract (web summary)

mTORC1 is a critical kinase that regulates cell growth and proliferation. Here the authors show that glutamine metabolism is sufficient to restore mTORC1 activity during prolonged amino acid starvation in an autophagy-dependent manner.

## Introduction

Living organisms are subjected to irregular nutrient supply and have evolved numerous adaptive mechanisms to appropriately respond to fluctuations in nutrient availability. Among the three macronutrients, the absence of a molecule that is specifically dedicated to the storage of amino acids distinguishes it from glucose and fatty acids. Although proteolysis contributes to amino acid balance during starvation, excessive proteolysis is detrimental to cell survival given that every cellular protein has a functional role^1, 2^. Therefore, amino acid metabolism and utilization require tight regulation to promote cell survival.

Mechanistic target of rapamycin complex 1 (mTORC1) is a serine/threonine protein kinase complex which plays a central role in the integration of nutritional and hormonal signals to regulate cellular biosynthesis and catabolic processes^3, 4^. In response to amino acids and growth factor activation, mTORC1 phosphorylates eukaryotic translation initiation factor 4E (eIF4E)-binding protein-1 (4EBP1) to relieve its inhibition on the assembly of translational pre-initiation complex^5^. Active mTORC1 also phosphorylates and activates p70S6K which in turn phosphorylates ribosomal protein S6 to enhance translation efficiency^6^. Thus, mTORC1 increases protein translation when amino acids are available. Amino acids are considered the most critical regulator of mTORC1 because growth factors alone cannot activate the kinase efficiently during amino acid deprivation^7^. In addition to its role in promoting anabolic pathways, mTORC1 represses catabolic processes like macroautophagy (hereafter referred to as autophagy) under nutrient-rich conditions. The kinase exerts its control over autophagy via Unc-51-like kinase 1 (ULK1) which is an upstream autophagy-related (ATG) protein^8^. In response to autophagy-inducing signals such as amino acid starvation, ULK1 forms a complex with multiple proteins including ATG13 to initiate autophagy. In its active state, mTORC1 associates with this complex, phosphorylates and inhibits ULK1 and ATG13 to repress autophagy^8^. Regulation of protein translation and autophagy by mTORC1 prevents futile cycles of protein synthesis and catabolism.

Autophagy is an evolutionarily conserved cellular process which involves the sequestration of intracellular proteins and organelles in autophagosomes and subsequent delivery of the cargo to lysosomes for degradation^9^. While low level of basal autophagy is important in the normal turnover of cellular proteins and organelles^9^, an elevation of autophagy is critical in sustaining amino acid levels to meet cellular demand when cells are starved of nutrients^10^. During amino acid starvation, autophagy supplies amino acids for the production of oxo-acids which replenish tricarboxylic acid cycle (TCA) intermediates via anaplerotic reactions^11^. Autophagy also provides amino acids for the translation of specific proteins such as metabolic enzymes and transporters, which constitute part of an amino acids restriction adaptation, to mitigate the nutritional stress^12^.

Glutamine is the most abundant free amino acid in the body, and accounts for ~20% of the total plasma free amino acid pool^13^. Numerous tissues particularly skeletal muscle synthesizes glutamine as an important carbon and nitrogen carrier, and free glutamine accounts for more than 60% of the tissue’s free amino acid pool^13^. Glutamine contains two nitrogen groups which are readily donated for non-essential amino acid (NEAA) synthesis, and its carbon skeleton is converted to α-ketoglutarate (α-KG) to support anaplerosis of the TCA cycle^14^. In addition to its abundance, the concentration of circulating glutamine is also labile. During starvation, increased proteolysis and amino acid catabolism boost the production and release of glutamine into circulation^15, 16^. In catabolic conditions such as systemic infection and severe injury, there is a sharp fall in the concentration of plasma glutamine, which exceeds the rate of decline of other amino acids^13^. Although the elevation of autophagy and increased glutamine production are adaptive responses to amino acid starvation, the crosstalk between glutamine and autophagy in the regulation of cell signaling and metabolism remains unclear.

We and others have previously reported that during amino acid starvation, autophagy is necessary for reactivation of mTORC1 signaling^17, 18^. It has been reported that autophagy-induced reactivation of mTORC1 is required for lysosomal recycling and restoration of protein translation^17^. Although the general consensus suggests that autophagy reactivates mTORC1 signaling by replenishing free amino acids, we and others have only detected a partially restored amino acid pool by autophagy^12, 18^, suggesting the existence of a more specific mechanism. In this study, we report that during amino acid starvation, glutamine is required for the reactivation of mTORC1 by autophagy. We report that supplementation of glutamine alone is sufficient to restore the activity and lysosomal localization of mTORC1 during prolonged amino acid starvation. Genetic or chemical inhibition of autophagy abolished the restorative effect of glutamine, suggesting that reactivation of mTORC1 is autophagy-dependent. Metabolomics analysis demonstrates that glutamine mediates mTORC1 reactivation specifically through its conversion to glutamate and restoration of NEAA pool. Consistently, mTORC1 reactivation is abolished when the conversion of glutamine to glutamate or subsequent transamination to NEAA is inhibited. Despite a persistent drop in essential amino acid (EAA) pool during amino acid starvation, glutamine and autophagy is sufficient to restore insulin sensitivity of mTORC1. Thus, glutamine metabolism plays a critical role in autophagy-dependent mTORC1 reactivation during amino acid starvation.

## Results

### Glutamine reactivates mTORC1 during amino acid starvation

**Fig. 1:**
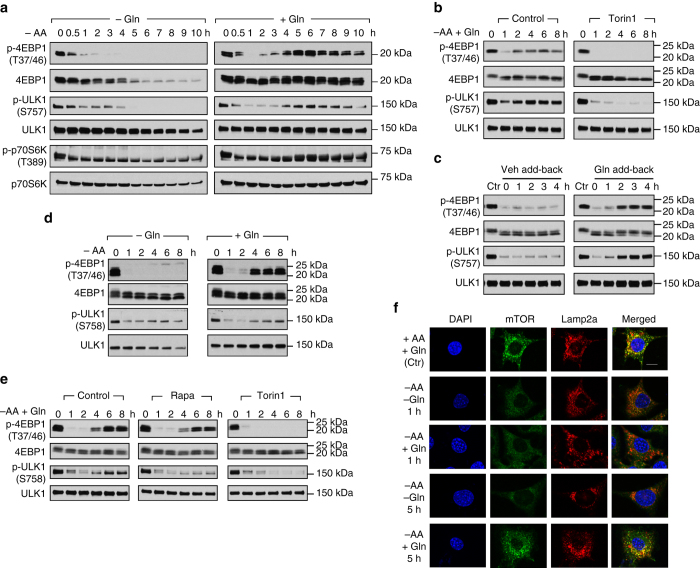
Glutamine reactivates mTORC1 signaling during amino acid starvation. **a** Wild-type MEFs were starved of amino acids with or without the supplementation of glutamine (4 mM) for the indicated durations. Changes in mTORC1 signaling were assessed by immunoblotting of p-4EBP1, p-ULK1, and p-p70S6K. **b** Wild-type MEFs were deprived of amino acids with the supplementation of glutamine (4 mM) for the indicated durations. Torin 1 (250 nM) was added upon induction of amino acid starvation to inhibit mTORC1. **c** Wild-type MEFs were deprived of amino acids for 5 h and cells were treated with H_2_O (vehicle add-back) or 4 mM glutamine (Gln add-back) for the indicated durations. **d** HepG2 cells were subjected to amino acid starvation in the absence or presence of glutamine (4 mM) for the indicated durations. **e** HepG2 cells were starved of amino acids with the supplementation of glutamine (4 mM) for the indicated durations. Rapamycin (50 nM) or Torin 1 (250 nM) was added upon induction of starvation to inhibit mTORC1. Changes in mTORC1 activity were assessed by immunoblotting of p-4EBP1 and p-ULK1. **f** Wild-type MEFs were starved of amino acids with or without the supplementation of glutamine (4 mM) for 1 or 5 h. Cells were then fixed and co-stained with antibodies specific for mTOR (*green*) and the lysosomal marker Lamp2a (*red*). The *scale bar* represents 10 µm

We first investigated the temporal dynamics of mTORC1 signaling of MEFs in response to amino acid starvation with or without supplementation of glutamine. In the absence of glutamine, amino acid starvation led to a progressive loss in the phosphorylation levels of 4EBP1 (T37/46), ULK1 (S757), and p70S6K (T389), direct downstream targets of mTORC1 (Fig. 1a). The marked loss in mTORC1 signaling persisted throughout the incubation period (Fig. 1a). In the presence of glutamine, amino acid starvation also led to an initial loss of mTORC1 signaling. However, glutamine induced a restoration of mTORC1 signaling in the later phase of amino acid starvation, as shown by the recovery of p-4EBP1 (T37/46), p-ULK1 (S757) and p-p70S6K (T389) (Fig. 1a). In order to verify that the restorative effect of glutamine on phosphorylation of these downstream targets was indeed mediated by mTORC1, we treated cells with Torin 1, a catalytic inhibitor of mTOR^6^. While glutamine restored p-4EBP1 (T37/46) and p-ULK1 (S757) during amino acid starvation, the effect was abolished by inhibition of mTORC1 (Fig. 1b). In order to evaluate whether glutamine is sufficient to restore mTORC1 signaling upon amino acid starvation, cells were starved of amino acids for 5 h followed by glutamine stimulation. We found that addition of glutamine alone is sufficient to reactivate mTORC1 signaling (Fig. 1c). The data suggest that glutamine restored mTORC1 signaling during amino acid starvation.

We next tested whether glutamine can restore mTORC1 signaling during amino acid starvation in another cell model. To this end, we subjected human hepatoma HepG2 cells to amino acid starvation in the absence or presence of glutamine. Amino acid starvation caused a progressive reduction of p-4EBP1 (T37/46) and p-ULK1 (S758), indicative of a loss in mTORC1 signaling (Fig. 1d). However, supplementation of glutamine restored mTORC1 signaling at the later time points (Fig. 1d), consistent with the findings in MEFs (Fig. 1a). Glutamine-mediated regain of p-ULK1 (S758) was abolished by Torin 1 and rapamycin (Fig. 1e), a specific mTORC1 inhibitor^6^. Restoration of p-4EBP1 (T37/46) was abolished by Torin 1, but was not affected by rapamycin, consistent with previous reports that p-4EBP1 (T37/46) is rapamycin-insensitive^19^. Collectively, the results suggest that glutamine restored mTORC1 signaling during amino acid limitation.

Given that localization of mTORC1 to the lysosomal surface is a key step in amino acid-induced mTORC1 activation^20^, we next investigated whether glutamine-mediated mTORC1 reactivation involves lysosomal localization of the kinase complex. To this end, we co-stained MEFs with antibodies specific to mTOR and the lysosomal-associated membrane protein 2 A (Lamp2a), a well-characterized lysosomal marker^20^, followed by confocal microscopy. Co-localization of mTOR with Lamp2a was not observed after an hour of amino acid starvation regardless of glutamine supplementation, as shown by a diffused cytoplasmic pattern of mTOR which did not overlap with Lamp2a (Fig. 1f), which correlated well with reduced mTORC1 signaling at 1 h after amino acid starvation (Fig. 1a). However, glutamine supplementation restored co-localization of mTOR and Lamp2a at 5 h after amino acid starvation (Fig. 1f), which coincided with reactivation of mTORC1 signaling (Fig. 1a). The data show that glutamine restored mTORC1 lysosomal localization and signaling during amino acid starvation, suggesting that restoration of amino acid balance may contribute to the recovery effect of glutamine on mTORC1.

### Autophagy is required for glutamine-induced mTORC1 signaling

**Fig. 2:**
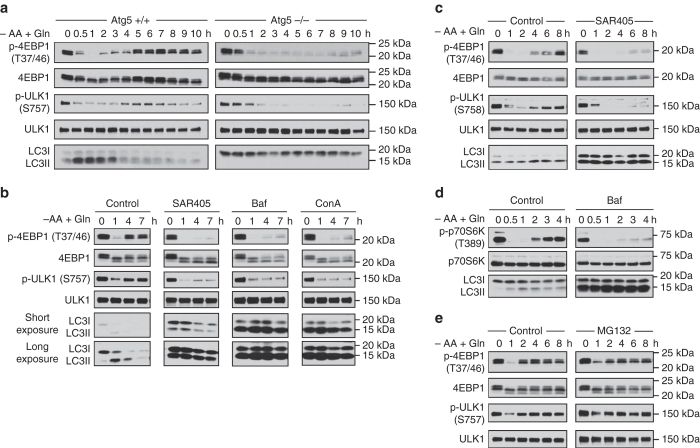
Autophagy is essential for glutamine-dependent restoration of mTORC1 signaling. **a** Wild-type (*Atg5*+/+) or *Atg5*-knockout (*Atg5*−/−) MEFs were subjected to amino acid starvation in the presence of glutamine (4 mM) for the indicated durations. **b** Wild-type MEFs were deprived of amino acids with the supplementation of glutamine (4 mM) for the indicated durations. Bafilomycin A1 (Baf, 200 nM), concanamycin A (ConA, 100 nM) or SAR405 (SAR, 5 µM) was added to inhibit autophagy. Changes in mTORC1 activity or autophagy were assessed by immunoblotting of p-4EBP1 and p-ULK1, or LC3, respectively. **c**, **d** HepG2 cells and C2C12 mouse myotubes were starved of amino acids as in **b** for the indicated durations. SAR405 (5 µM) and bafilomycin A1 (200 nM) was added to inhibit autophagy in HepG2 cells and C2C12 myotubes, respectively. Changes in mTORC1 activity were assessed by immunoblotting of p-4EBP1 and p-ULK1 (for HepG2 cells) or p-p70S6K (for C2C12 myotubes), and changes in autophagy was evaluated by immunoblotting of LC3. **e** Wild-type MEFs were deprived of amino acids with the supplementation of glutamine (4 mM) for the indicated durations. MG132 (10 µM) was added to inhibit proteasome function. Changes in mTORC1 activity were assessed by immunoblotting of p-4EBP1 and p-ULK1

We observed that the effect of glutamine on mTORC1 reactivation occurred at a later phase (after about 4 h) of amino acid starvation, which coincided with the initiation of autophagic proteolysis, as shown by the reduction of LC3II band (Fig. 2a). The results suggest that autophagy might play a role in glutamine-induced mTORC1 reactivation in MEFs. Consistent with this notion, genetic ablation of *Atg5* in MEFs (*Atg5*−/−) abolished the restorative effect of glutamine on mTORC1 signaling, as demonstrated by a persistent loss of p-4EBP1 (T37/46) and p-ULK1 (S757) (Fig. 2a). In order to exclude possible confounding factors of long-term genetic modification in *Atg5*−/− MEFs, we turned to chemical inhibitors of autophagy and lysosomal function. Pharmacological inhibition of autophagy by blocking autophagosome initiation (with SAR405) or lysosomal function (with bafilomycin A1, Baf; or concanamycin A, ConA), consistently abolished glutamine-induced mTORC1 reactivation in MEFs (Fig. 2b). Similarly, inhibition of autophagosome initiation abolished glutamine-dependent mTORC1 reactivation in human hepatoma HepG2 cells during amino acid starvation (Fig. 2c). Consistent with our previous observation, inhibition of lysosomal function abolished mTORC1 reactivation in differentiated C2C12 mouse myotubes (Fig. 2d). Although the proteasome was shown to contribute to amino acids homeostasis^21^, inhibition of proteasome function with MG132 did not interfere with glutamine-induced mTORC1 reactivation in MEFs (Fig. 2e). Thus, autophagy is essential for glutamine-induced mTORC1 signaling, a mechanism which is well-conserved in various cell models tested.

### Glutamine metabolism is essential for mTORC1 reactivation

**Fig. 3:**
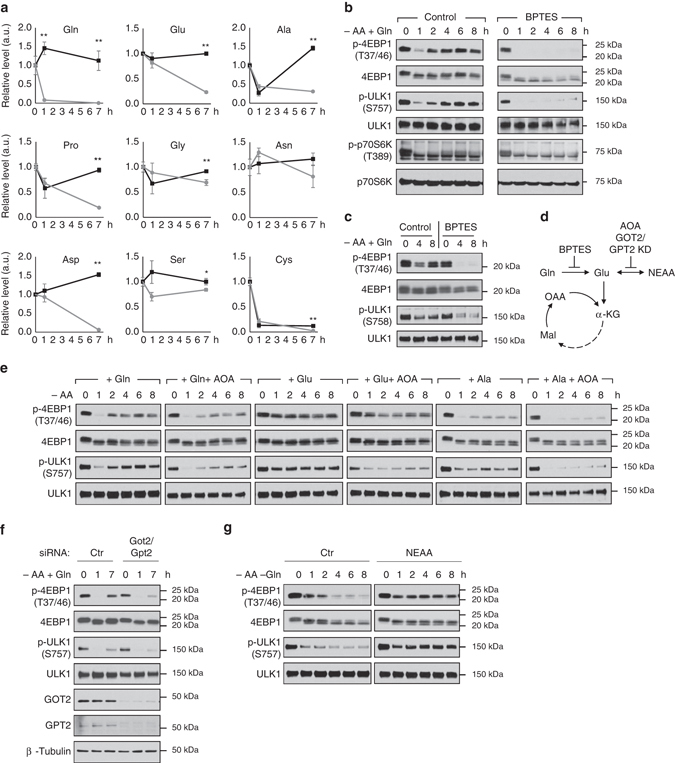
Glutamine metabolism is essential for mTORC1 reactivation during amino acid starvation. **a** Wild-type MEFs were starved of amino acids with (*black line*) or without (*gray line*) the supplementation of glutamine (4 mM) for the indicated durations. Individual non-essential amino acid (NEAA) levels were measured from cell lysates by GC-TOF-MS. Data are expressed as the fold change of control cells (unstarved control, 0 h). Data are the mean ± SEM of *n* = 4–5, *P≤0.05; **P≤0.01 (between the groups with and without glutamine supplementation at the same time point via Student’s *t*-test). **b** Wild-type MEFs or **c** HepG2 cells were deprived of amino acids with the supplementation of glutamine (4 mM) for the indicated durations in the absence or presence of BPTES (10 µM). Changes in mTORC1 activity were assessed by immunoblotting of p-4EBP1 and p-ULK1. **d** Schematic diagram of glutamine metabolism. Glutamine is converted to glutamate by glutaminase, which is inhibited by BPTES. Glutamate undergoes transamination (which is inhibited by amino-oxyacetate, AOA, or knockdown of GOT2/GPT2), to produce non-essential amino acids (NEAA) and α-KG (a TCA cycle intermediate). **e** Wild-type MEFs were starved of amino acids with the supplementation of 4 mM glutamine, glutamate or alanine in the presence or absence of AOA (5 mM). **f** Wild-type MEFs were subjected to siRNA-mediated knockdown of GOT2 and GPT2, and cells were deprived of amino acids with the supplementation of glutamine (4 mM) for the indicated durations. **g** Wild-type MEFs were starved of amino acids with or without the supplementation of NEAA for the indicated durations. Changes in mTORC1 activity were assessed by immunoblotting of p-4EBP1 and p-ULK1

To study the mechanism underlying the restorative effect of glutamine on mTORC1 signaling during amino acid starvation, we evaluated the abundance of NEAAs in MEFs with metabolomics. Among the 9 NEAAs detected, glutamine displayed the greatest reduction (92%) in the first hour of amino acid starvation (Fig. 3a). The sharp depletion of glutamine is associated with a milder rate and/or magnitude of decline in glutamate, alanine, and aspartate, the major amino acids which are directly transaminated to support NEAAs synthesis^14^. Given that glutamine depletion precedes that of glutamate, alanine and aspartate, it suggests that glutamine may be the preferred source of nitrogen for maintenance of NEAA pool during amino acid starvation. Consistently, we found that supplementation of glutamine restored or even sustained most of the intracellular NEAAs during amino acid deprivation (Fig. 3a). The restorative effect of glutamine on NEAAs appeared to be specific to its ability to donate nitrogen groups as the level of cysteine, a sulfur-containing NEAA is not recovered by glutamine (Fig. 3a).

We next asked whether glutamine catabolism, which is necessary for its nitrogen-donating function, is required for its restorative effect on mTORC1 signaling. The first step of glutamine catabolism involves the conversion of glutamine to glutamate by glutaminase (Fig. 3d). We found that the ability of glutamine to restore p-4EBP1 (T37/46), p-ULK1 (S757), and p-p70S6K (T389) during amino acid starvation in MEFs was abolished by BPTES (Fig. 3b), a selective inhibitor of glutaminase^22^. Similarly, inhibition of glutaminase in HepG2 cells abolished the restorative effect of glutamine on mTORC1 signaling (Fig. 3c). Given that the preceding experiments were performed in EBSS, we verified the results by treating cells in DMEM. We found that glutamine-mediated reactivation of mTORC1 signaling was abolished by autophagy or glutaminase inhibition in DMEM (Supplementary Fig. 1), consistent with previous results obtained in EBSS. Thus, conversion of glutamine to glutamate is required for mTORC1 reactivation during amino acid starvation.

The first product of glutamine catabolism is glutamate which acts as the primary nitrogen donor to support amino acid balance via transamination reactions (Fig. 3d). We next studied whether glutamine mediates its restorative effect on mTORC1 signaling by generating glutamate to restore NEAA balance. We treated MEFs with amino-oxyacetate (AOA), an inhibitor of transamination^14^, and observed that glutamine-induced mTORC1 reactivation was severely impaired (Fig. 3e). Supplementation of glutamate recapitulated the rescuing effect of glutamine on mTORC1 reactivation, which was similarly impaired in the presence of AOA (Fig. 3e), suggesting transamination is required for its effect. Alanine is another amino acid which can act as a systemic carbon and nitrogen carrier like glutamine. In contrast to glutamine, we observed a modest effect on restoration of mTORC1 signaling with supplementation of alanine, suggesting the primary role of glutamine. Nonetheless, the effect of alanine was also dependent on transamination given that its effect was abolished by AOA (Fig. 3e). In order to address the non-specific effects of AOA, we performed siRNA-mediated knockdown of both glutamic-oxaloacetic transaminase 2 (GOT2) and glutamic-pyruvic transaminase 2 (GPT2) to inhibit transamination. Consistent with our results obtained with AOA, knockdown of both GOT2 and GPT2 impaired glutamine-mediated restoration of mTORC1 signaling (Fig. 3f). Moreover, a mixture of NEAA (Ala, Pro, Gly, Asn, Asp, and Ser) was sufficient to reactivate mTORC1 in the absence of other amino acids (Fig. 3g). Thus, the restorative effect of glutamine on mTORC1 signaling requires the conversion of glutamine to glutamate which in turn sustain the NEAA pool via transamination.

Both glutaminolysis and glutamate dehydrogenase reactions produce ammonia which can modulate autophagy^23, 24^, or act as a nitrogen donor to reactivate mTORC1. In order to evaluate the role of ammonia in glutamine-mediated mTORC1 reactivation, we measured ammonia concentration and found that glutamine did not significantly alter intracellular ammonia concentrations during amino acid starvation (Supplementary Fig. 2A). However, glutamine increased extracellular ammonia concentration, suggesting that ammonia which is produced by glutamine catabolism is predominantly released into the extracellular medium (Supplementary Fig. 2A). Moreover, both time- and dose-dependent experiments did not show that exogenous supplementation of ammonia restored mTORC1 signaling during amino acid starvation (Supplementary Fig. 2B and C). Although entry of ammonia could potentially alter intracellular pH, our data is consistent with previous reports that exogenous ammonia does not activate mTORC1^23, 25^. Therefore, glutamine (via its conversion to glutamate) is the primary nitrogen donor to promote mTORC1 reactivation, and exogenous ammonia could not replace the role of glutamine.

### Glutamine restores mTORC1 signaling independent of EAAs

**Fig. 4:**
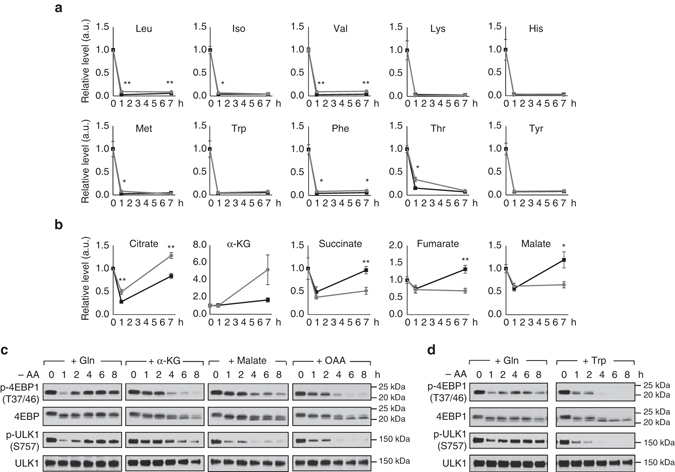
Glutamine reactivates mTORC1 signaling independent of EAAs. **a**, **b** Wild-type MEFs were starved of amino acids with (*black line*) or without (*gray line*) the supplementation of glutamine (4 mM) for the indicated durations. Individual essential amino acid **a** and TCA cycle intermediate **b** levels were measured from cell lysates by GC-TOF-MS. Data are expressed as the fold change of control cells (unstarved control, 0 h). Data are the mean ± SEM of *n* = 4–5, *P≤0.05; **P≤0.01 (between the groups with and without glutamine supplementation at the same time point via Student’s *t*-test). **c** Wild-type MEFs were subjected to amino acid starvation with the supplementation of 4 mM glutamine, dimethyl-α-KG, malate or OAA for the indicated durations. **d** Wild-type MEFs were starved of amino acids with the supplementation of glutamine (4 mM) or tryptophan (4 mM) for the indicated durations. Changes in mTORC1 activity were assessed by immunoblotting of p-4EBP1 and p-ULK1

The previous results demonstrate that glutamine mediates reactivation of mTORC1 via its production of glutamate which in turn sustains the NEAA pool (Fig. 3). Consistent with this notion, we observed that glutamine induced a remarkable rebound or maintenance of the NEAA abundance including glutamate, alanine, proline, glycine, aspartate, and serine in MEFs (Fig. 3a). In contrast, glutamine failed to restore any of the EAA levels (Fig. 4a). Moreover, addition of tryptophan (an EAA) did not restore mTORC1 signaling during amino acid starvation (Fig. 4d). Thus, restoration of NEAAs, but not EAAs contributes to glutamine-induced mTORC1 reactivation during amino acid starvation.

While transamination of glutamate produces other NEAAs by donating its amino group, its carbon skeleton is converted to α-KG, an important TCA cycle intermediate. Thus, catabolism of glutamine to glutamate, which is then transaminated to α-KG, is important for maintaining energy homeostasis^22^. We then evaluated whether the anaplerotic role of glutamine contributes to mTORC1 signaling in MEFs. Metabolomics analysis revealed that during amino acid starvation, α-KG is sustained when glutamine is supplemented, and is elevated when glutamine is absent (Fig. 4b). Given the metabolic profile did not correlate with mTORC1 reactivation (Fig. 4b), the data suggest that α-KG does not contribute to the restorative effect of glutamine. Consistently, supplementation of α-KG did not restore mTORC1 signaling (Fig. 4c). Although the metabolic profile of fumarate and malate resembles the dynamics of NEAAs such as alanine (Figs. 3a and 4b), TCA cycle intermediates distal to α-KG also did not appear to support mTORC1 reactivation, given that supplementation of malate and oxaloacetate (OAA) did not restore mTORC1 signaling (Fig. 4c). Glutamine-mediated production of cellular glutamate and aspartate can alter cell volume which has been shown to alter autophagy and mTORC1 activity^26–28^. We evaluated cell size under amino acid-starved conditions in the absence or presence of glutamine, but did not detect any significant alteration in cell size (Supplementary Fig. 3A). We also considered the possibility that glutamine-derived glutamate and aspartate supports the malate-aspartate shuttle to reactivate mTORC1, through the restoration of glycolytic NADH oxidation. Inhibition of the malate-aspartate shuttle by malate dehydrogenase 2 (MDH2) inhibitor LW6^29^, which led to an increase in lactate production, was without effect on glutamine-induced mTORC1 reactivation (Supplementary Fig. 3B). Moreover, numerous glycolytic intermediates remained unaltered in response to glutamine treatment (Supplementary Fig. 3C). The results led us to conclude that glutamate, but not α-KG, mediates the restorative effects of glutamine on mTORC1 reactivation. However, we observed that α-KG, malate and OAA transiently sustained mTORC1 signaling in the first 2 h of amino acid starvation (Fig. 4c). We speculate that these keto acids contribute carbon source to NEAAs synthesis, or spare NEAA catabolism for anaplerosis, to temporarily sustain mTORC1 signaling. However, in the absence of a nitrogen source, the keto acids could not rescue the loss of mTORC1 activity during prolonged amino acid starvation.

### Glutamine and autophagy sustain insulin-activated mTORC1

**Fig. 5:**
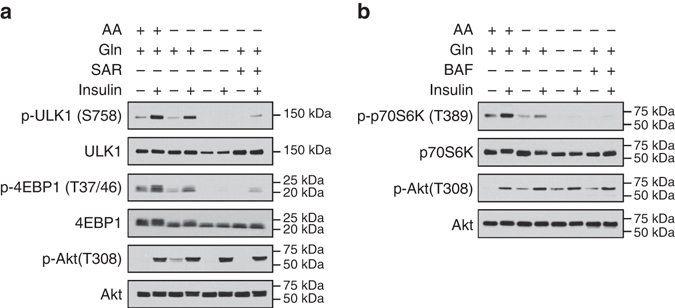
Glutamine and autophagy sustain insulin-induced mTORC1 signaling during amino acid starvation. **a** HepG2 cells were pre-incubated for 30 min in EBSS with amino acids. Cells were then starved of amino acids in the presence or absence of glutamine (4 mM) with or without SAR405 (5 µM) for 5 h, followed by insulin (50 nM) stimulation for an additional 30 min. Changes in mTORC1 activity were assessed by immunoblotting of p-4EBP1 and p-ULK1. Insulin stimulation was assessed by immunoblotting of p-Akt. **b** C2C12 myotubes were pre-incubated for 30 min in EBSS with amino acids. Myotubes were then starved of amino acids in the presence or absence of glutamine (4 mM) with or without bafilomycin A1 (200 nM) for 5 h, followed by insulin (50 nM) stimulation for an additional 30 min. Changes in mTORC1 activity were assessed by immunoblotting of p-p70S6K. Insulin stimulation was assessed by immunoblotting of p-Akt

Although amino acids and growth factors can activate mTORC1 via separate pathways, amino acids is a permissive factor for the action of growth factors^7^. Amino acids promote the translocation of mTORC1 to the lysosomal surface to interact with Rheb, which transduce growth factor signals to activate mTORC1^3^. Our data thus far demonstrated that glutamine cooperates with autophagy to mediate mTORC1 reactivation via the generation of NEAAs. However, the amino acid pool restoration is incomplete, given that EAAs remained almost undetectable (Fig. 4a). We next assessed whether glutamine-mediated reactivation of mTORC1, despite an incomplete restoration of the amino acid pool, is able to maintain insulin-induced mTORC1 signaling. When HepG2 cells were stimulated with insulin in the presence of amino acids, there was an increase in p-ULK1 (S758) and p-4EBP1 (T37/46) as expected (Fig. 5a). In the absence of amino acids, supplementation of glutamine alone was able to sustain insulin-induced mTORC1 signaling. However, removal of glutamine abolished the insulin effect, despite an intact p-Akt (T308) induction by insulin (Fig. 5a). Similar results were obtained in C2C12 myotubes, in which glutamine sustained insulin-induced p-p70S6K (T389) during amino acid starvation (Fig. 5b). Thus, the restorative effect of glutamine on mTORC1 signaling during amino acid starvation is sufficient to support the input of signals from growth factors, consistent with the ability of glutamine to restore proper lysosomal localization of mTORC1 (Fig. 1f).

Given that restoration of mTORC1 activation by glutamine is autophagy-dependent (Fig. 2), we next assessed whether autophagy is necessary for glutamine to sustain insulin-activated mTORC1 during amino acid starvation. Although glutamine was able to maintain insulin signaling to mTORC1 during amino acid starvation, we found that the effects were abolished when autophagy was inhibited by SAR405 and Baf in HepG2 cells and C2C12 myotubes, respectively (Fig. 5a and b). Autophagy inhibitors did not interfere with the ability of growth factor to stimulate p-Akt (T308), suggesting that glutamine and autophagy cooperate to regulate the amino acid pathway upstream of mTORC1. Thus, crosstalk between glutamine and autophagy supports mTORC1 reactivation and insulin sensitivity during amino acid starvation.

## Discussion

During starvation, the cellular amino acid pool is maintained predominantly by proteolysis, which contributes free amino acids for translation of specific stress-induced proteins, as well as carbon skeletons for energy production^10, 12^. Therefore, proteolysis and protein translation has to be coordinated to support cell survival. Autophagy is a major cellular proteolytic process which degrades cellular constituents and releases free amino acids as part of a starvation adaptation^9^. Although mTORC1 signaling is repressed during amino acid starvation to activate autophagy and reduce translation to restore amino acid balance, we and others have shown that autophagy can contribute amino acids to restore mTORC1 activity^17, 18^. Autophagy-mediated restoration of mTORC1 in turn induces autophagy termination and reformation to lysosomes which completes the feedback loop^17^. Although it is tempting to envisage that autophagy restores the amino acid pool to reactivate mTORC1, we and others have detected only a partial restoration of amino acids abundance by autophagy^12, 18^, hinting the existence of a more precise mechanism. During starvation, there is an increase in the synthesis and release of glutamine which serves as the primary nitrogen carrier in the blood^15, 16^. However, the role of glutamine in starvation-induced signaling and adaptation remains largely unknown.

In this study, we show that glutamine is required for autophagy-induced mTORC1 reactivation during amino acid starvation. Although autophagy releases free amino acids during amino acid deprivation, the level of replenishment is substantially incomplete. In response to amino acid starvation, autophagy failed to restore the levels of amino acids beyond 50% in yeast, for thirteen of the nineteen proteinogenic amino acids detected^12^. In mouse myotubes, 80% of all EAA remained undetectable during amino acid starvation, despite an induction of autophagy^18^. Our current results demonstrate that supplementation of glutamine, an amino acid which is selectively produced during starvation, is required to restore mTORC1 signaling and lysosomal localization during amino acid starvation (Fig. 1). However, the restorative effect of glutamine on mTORC1 signaling requires autophagy, as genetic or pharmacological inhibition of autophagosome and/or lysosomal function abolished glutamine-induced mTORC1 reactivation (Fig. 2). Consistent with our previous findings in myotubes^18^, proteasome inhibition did not interfere with mTORC1 reactivation in MEFs (Fig. 2e), suggesting that autophagy is the predominant proteolytic system which supports mTORC1 signaling. Thus, glutamine and autophagy constitute a starvation-specific signaling program to reactivate mTORC1.

When MEFs were deprived of amino acids, we found that there was a decrease in both NEAAs and EAAs (Figs. 3a and 4a), despite the induction of autophagy. The failure of autophagy to sustain the amino acid pool is consistent with the decline in mTORC1 signaling (Fig. 1a). It is interesting to note that among all the NEAAs, glutamine displayed the sharpest drop in abundance which preceded others, presumably in a vain attempt to restore other NEAAs (Fig. 3a). Consistent with this notion, glutamine supplementation restored or even maintained most of the intracellular NEAAs during amino acid starvation (Fig. 3a). It is recognized that glutamine is an important carrier for shuttling of nitrogen among tissues, due to its capacity to readily donate amide and amino nitrogen^13^. Our data here provided evidence that glutamine is the preferred intracellular nitrogen donor during amino acid starvation, which corroborated its role as a nitrogen donor.

Although mTORC1 is activated by amino acid availability, certain amino acids such as leucine appear to mediate a greater control^3^. It has been proposed that leucine being an EAA, its concentration in the blood varies according to its dietary intake, and can thus signal amino acids availability to mTORC1^30^. It has been reported that EAAs, particularly leucine play a critical role in the activation of mTORC1 by glutamine^31^. The model describes uptake of extracellular glutamine and its subsequent efflux in exchange for influx of EAA including leucine, which in turn activates mTORC1^31^. Although deprivation of extracellular EAAs precluded this mechanism in our model, we considered the potential contribution of EAAs, given that glutamine is known to have a leucine-sparing effect^32^. Nonetheless, glutamine did not restore any of the EAAs in MEFs (Fig. 4a), despite a pronounced reactivation of mTORC1 during amino acid starvation (Fig. 1a). The results suggest that glutamine-mediated restoration of NEAAs is sufficient to reactivate mTORC1 in MEFs. Nonetheless, the marked reduction of EAAs in the whole-cell lysate does not exclude the possibility that autophagy might lead to a localized high EAAs concentration in the lysosome, which could contribute to Gln-dependent mTORC1 reactivation. Glutaminolysis involves the conversion of glutamine to glutamate, which is the major amino acid involved in the synthesis of NEAA via transamination. When glutaminolysis or transamination is inhibited, the restorative effect of glutamine on mTORC1 activation is abolished (Fig. 3b–f), suggesting that both steps are essential for the effect of glutamine. Supplementation of glutamate, the product of glutaminolysis, reactivated mTORC1 and this effect is also dependent on transamination (Fig. 3e). Although synthesis and release of alanine, like glutamine, is also elevated in response to starvation^15^, we observed a modest effect on mTORC1 reactivation when cells were treated with alanine (Fig. 3e). We also observed that exogenous ammonia could not replace the role of glutamine in mTORC1 reactivation (Supplementary Fig. 2B and C), consistent with previous reports that ammonia does not activate mTORC1^23, 25^. This suggests that glutamine, which is readily transported into the cells^31^, and preferentially catabolized during amino acid starvation (Fig. 3a), may be a relatively more reliable nitrogen transporter/donor than ammonia which relies on diffusion for cell entry^23^. Moreover, a mixture of NEAA (without glutamine and glutamate) is sufficient to restore mTORC1 signaling during amino acid starvation (Fig. 3g). Thus, the restorative effect of glutamine on mTORC1 signaling requires the conversion of glutamine to glutamate, which in turn sustains the NEAA pool via transamination.

Our results demonstrate that glutamine metabolism, but not glutamine per se, mediates mTORC1 reactivation under amino acid-starved conditions. This is in contrast with a recent study showing that glutamine, but not glutamate, stimulates mTORC1 in RagA/B knockout MEFs via ADP ribosylation factor 1 (Arf1)^33^. Thus, there are distinct mechanisms that specifically drive acute glutamine-stimulated mTORC1 and glutamine-induced mTORC1 reactivation under prolonged starvation. Indeed, we found that glutamine-mediated mTORC1 reactivation was not perturbed by inhibition of Arf1 (data not shown). In addition to its role as a nitrogen donor, glutamine serves as a major anaplerotic precursor through its catabolism to α-KG, which has been shown to activate mTORC1 signaling^34^. However, we excluded the direct role of α-KG in our model because temporal changes in α-KG levels did not correlate with the dynamics of mTORC1 signaling (Figs. 1a and 4b), and supplementation of α-KG did not have any discernable effect on mTORC1 signaling (Fig. 4c). This discrepancy may be due to differences in experimental conditions. The report by Duran et al.^34^ demonstrated that glutamine-derived α-KG is necessary for leucine-induced mTORC1 stimulation (within 15 min) after an acute (1 or 2 h) amino acid starvation. In contrast, we focused on mTORC1 reactivation by glutamine alone (without leucine) during prolonged (0–8 h) amino acid starvation. Thus, different mechanisms is likely involved in mTORC1 activation: glutamine-derived alpha-ketoglutarate mediates leucine-induced mTORC1 signaling during acute amino acid starvation, whereas glutamine-derived NEAA mediates autophagy-dependent mTORC1 reactivation during prolonged amino acid starvation. We observed that glutamine restored the levels of TCA cycle intermediates downstream of α-KG (succinate, fumarate, and malate). However, supplementation of distal TCA cycle intermediates including malate or OAA in MEFs did not restore mTORC1 signaling, suggesting that glutamine-dependent anaplerosis is not responsible for the reactivation of mTORC1 during starvation.

Although amino acids and growth factors activate mTORC1 via distinct pathways, amino acid sufficiency is required for growth factor to activate mTORC1. Growth factors activate mTORC1 via RHEB which is anchored at the lysosome, and mTORC1 is only recruited to the lysosome by the Rag heterodimer upon amino acid stimulation^20^. Our results demonstrated that in the complete absence of amino acids, supplementation of glutamine alone is sufficient to induce mTORC1 lysosomal localization which restore its sensitivity to insulin stimulation (Figs. 1f and 5). Thus, during amino acid starvation, glutamine alone is able to restore mTORC1 activation and sensitivity to insulin. Multiple cancer cell types have been found to display increased rates of glutamine uptake and catabolism, and some cannot survive without exogenous glutamine (termed glutamine-addicted cells), effects which are mainly ascribed to its ability to sustain the TCA cycle^14^. In the clinics, anabolic trophic effects of glutamine supplementation in catabolic diseases have also been reported^35^. Our findings may provide an explanation for the anabolic effects of glutamine, in addition to its anaplerotic role. Nonetheless, the restorative effect of glutamine on mTORC1 and insulin sensitivity requires autophagy, consistent with previous observations that autophagy contribute to amino acid balance during starvation^12, 18^. Thus, crosstalk between glutamine metabolism and autophagy sustains input of growth factor signals to mTORC1 during amino acid starvation (Fig. 5).

Numerous models of mTORC1 activation by glutamine and growth factors describes the obligatory role of EAAs^3, 31^. Our findings provides a new mechanism for mTORC1 reactivation during amino acid starvation. While autophagy is required for mTORC1 reactivation during complete amino acid starvation, autophagy alone is insufficient to replenish the amino acid pool to reactivate mTORC1. Our results show that glutamine cooperates with autophagy to reactivate mTORC1, through its conversion to glutamate and subsequent transamination to NEAAs. Restoration of mTORC1 signaling by glutamine and autophagy is sufficient to confer insulin sensitivity of mTORC1, even in the absence of intracellular EAAs. Given that proteolysis and amino acids catabolism increase glutamine synthesis^15, 16^, the present data suggest that extracellular glutamine may function as specific starvation-induced nutrient signal to regulate mTORC1.

## Methods

### Materials

Dilution at each antibodies was used are stated after the catalog number of each commercial antibodies. Antibodies against p-ULK1 (Ser757) (#6888; 1:1000), ULK1 (#8054; 1:1000), p-4EBP1 (Thr37/46) (#2855; 1:1000), 4EBP1 (#9644; 1:1000), p-p70S6K (Thr389) (#9234; 1:1000), p70S6K (#2708; 1:1000), p-Akt (Thr308) (#9275; 1:1000), Akt (#4691; 1:1000), LC3A (#4599; 1:1000), LC3B (#3868; 1:1000), mTOR (#2983; 1:100), β-tubulin (#2128; 1:1000) and Alexa 488 anti-rabbit secondary (#4412; 1:200) antibodies were from Cell Signaling Technology (Beverly,MA). Anti-LAMP2 antibody (ab13524; 1:100) was from Abcam (Cambridge, UK). Alexa 555 anti-rat secondary antibody (A21434; 1:120) was from Thermo Fisher Scientific (Waltham, MA). GOT2 antibody (14800-1-AP; 1:1000) from ProteinTech (Rosemont, IL), GPT2 (sc-398383; 1:500) from Santa Cruz (Dallas, TX). Rapamycin, bafilomycin A1, concanamycin A and MG132 were from Cayman Chemical (Ann Arbor, MI). Torin 1 was from Tocris Bioscience (Bristol, UK). SAR405 was from ApexBio Technology (Houston, TX). Bis-2-(5-phenylacetamido-1,3,4-thiadiazol-2-yl)ethyl sulfide (BPTES), amino-oxyacetate hemichloride (AOA), dimethyl-αKG, malate and oxaloacetate were from Sigma-Aldrich (St Louis, MO).

### Cell culture

Wild-type (*Atg5*+/+) and *Atg5*-knockout (*Atg5*−/−) mouse embryonic fibroblasts (MEFs) were kindly provided by Noboru Mizushima (Tokyo Medical and Dental University, Tokyo, Japan) and have been previously described^10^. Human HepG2 hepatoma cells and mouse C2C12 myoblasts were obtained from American Type Culture Collection (Manassas, VA). All cells were maintained in DMEM (D7777, Sigma-Aldrich, St Louis, MO) supplemented with 3.7 g l^−1^ NaHCO_3_, 25 mM HEPES, 10% fetal bovine serum (FBS), 100 U ml^−1^ penicillin and 100 μg ml^−1^ streptomycin. Cells were maintained in a 5% CO_2_ humidified incubator. For C2C12 myoblasts, media was switched to differentiation media (DMEM supplemented with 2% horse serum, 100 U ml^−1^ penicillin and 100 μg ml^−1^ streptomycin) over 3–5 days to initiate the differentiation of myoblasts to myotubes.

### Amino acid starvation and siRNA treatment

For amino acid starvation, cells were first switched to Earle’s balanced salt solution (EBSS) supplemented with 25 mM glucose, 1X MEM amino acids solution (Life Technologies, Carlsbad, CA), 4 mM glutamine, 1X MEM vitamin solution (Life Technologies), 0.2% bovine serum albumin (BSA), 3.7 g l^−1^ NaHCO_3_, 25 mM HEPES, 100 U ml^−1^ penicillin and 100 µg ml^−1^ streptomycin. The pH of media was adjusted and cells were treated at pH 7.4. Cells were pre-incubated in EBSS with amino acids for 30 min prior to starvation. Amino acid starvation was performed by selective exclusion of 1X MEM amino acids from EBSS (unless stated otherwise) with or without the supplementation of 4 mM glutamine as indicated. For siRNA treatment, MEFs were reverse transfected for 48 h with a combination of 100 pmol Got2 DsiRNA (IDT, mm.Ri.Got2.13.3) and 100 pmol Gpt2 DsiRNA (IDT, mm.Ri.Gpt2.13.3) or control DsiRNA (IDT. Cat. No. 51-01-14-04) before treatment. Cell culture medium is supplemented with Ala and Asp (at 0.1 mM each) during the 48 h siRNA treatment. For NEAA supplementation, the NEAA mixture consists of Ala, Pro, Gly, Asn, Asp, and Ser, each at 0.1 mM (based on the concentrations of 1× MEM Non-Essential Amino Acids Solution, Gibco 11140050).

### Immunoblotting

Cells were homogenized in ice-cold lysis buffer containing 137 mM NaCl, 2.7 mM KCl, 1 mM MgCl_2_, 1% Triton X-100, 10% glycerol, 20 mM Tris pH 7.8, 1 mM EDTA, 1 mM DTT, 5 mM β-glycerophosphate, 5 mM NaF, 5 mM Na_3_VO_4_, 5 mM Na_4_P_2_O_7_, and 1% Halt protease inhibitor cocktail (Life Technologies, Carlsbad, CA). Lysates were subjected to brief sonication, centrifuged at 13,000×*g* for 10 min to remove insoluble cell debris and the supernatant was diluted in Laemmli sample buffer. Proteins (30 μg per well) were separated in 10 or 6–15% gradient SDS-PAGE gels and transferred to PVDF membranes (Bio-Rad Laboratories, Hercules, CA). Membranes were blocked with 5% non-fat milk, incubated overnight with primary antibodies, washed, and then incubated with HRP-linked secondary antibodies (#7074 rabbit and #7076 mouse, at 1:2000 to 1:10,000 dilution) from Cell Signaling Technology (Beverly,MA). Proteins were then visualized by chemiluminescence. Wider crop of representative blots in the main figures are presented in Supplementary Fig. 4.

### Immunofluorescence and flow cytometry

MEFs were seeded on 12 mm glass coverslips, left to attach overnight and treated as indicated. Cells were fixed in 4% paraformaldehyde for 20 min, washed three times in PBS, permeabilized in 0.05% Triton X-100 in PBS for 5 min and blocked in blocking buffer (5% FBS and 2% BSA in PBS) for 1 h. Cells were then incubated in primary antibodies overnight, followed by three washes in PBS and incubation in secondary antibodies (anti-rabbit Alexa 488 or anti-rat Alexa 555) for 1.5 h. Samples were then mounted in VECTASHIELD anti-fade mounting medium with DAPI (Vector Laboratories, Burlingame, CA), and imaged on an Olympus FV1200 confocal microscope at ×60 magnification. For analysis of cell size, MEFs were collected after treatment and subjected to flow cytometry using BD FACsCantoTM II system. FACs data were processed using FlowJo software and cell size was determined by forward scatter height (FSC-H).

### Analysis of metabolites

For metabolomics analysis, MEFs were seeded on 100 mm plates, left to attach overnight and treated as indicated. Cell culture medium was removed and cells were rinsed with PBS and homogenized in acetonitrile, isopropanol and water (ratio of 3:3:2), and analyzed by automatic liner exchange/cold injection GC-TOF mass spectrometry at West Coast Metabolomics Centre (Davis, CA) for measurement of metabolites. For evaluation of cellular ammonia and lactate, MEFs were treated as indicated, conditioned media was collected and cells were rinsed once with PBS and snap frozen in liquid nitrogen. Extracellular and intracellular ammonia were quantified with an ammonia assay kit (Biovision #K370-100), and intracellular lactate was determined with a lactate assay kit (Biovision #K607-100) following the recommended manufacturer’s protocol.

### Statistical analysis

Data are presented as the mean ± SEM. Differences between groups were determined by Student’s *t*-Test. Grubb’s test was used to detect and exclude outliers. Statistical significance was accepted at *P*<0.05. Experiments were replicated twice for Fig. 1a–d, Fig. 2a and b, Fig. 3b, c and g, Fig. 4c, and Fig. 5a, and replicated once for Fig. 1e, Fig. 2c–e, Fig. 3e and f, Fig. 4d, Fig. 5b, Supplementary Fig. 1, Supplementary Fig. 2B and C and Supplementary Fig. 3B.

### Data availability

All relevant data are available from the corresponding author on reasonable request.

## Electronic supplementary material

Supplementary Information
