# Supplementary material for: Glutamine metabolism regulates autophagy-dependent mTORC1 reactivation during amino acid starvation
Source: Nat Commun. 2017 Aug 24;8:338. doi: 10.1038/s41467-017-00369-y (PMC5569045; doi:10.1038/s41467-017-00369-y)
Supplement: Supplementary file 1 — Supplementary Information [file 41467_2017_369_MOESM1_ESM.pdf]

Description of Supplementary Files

Title: Supplementary Information

Description: Supplementary Figures

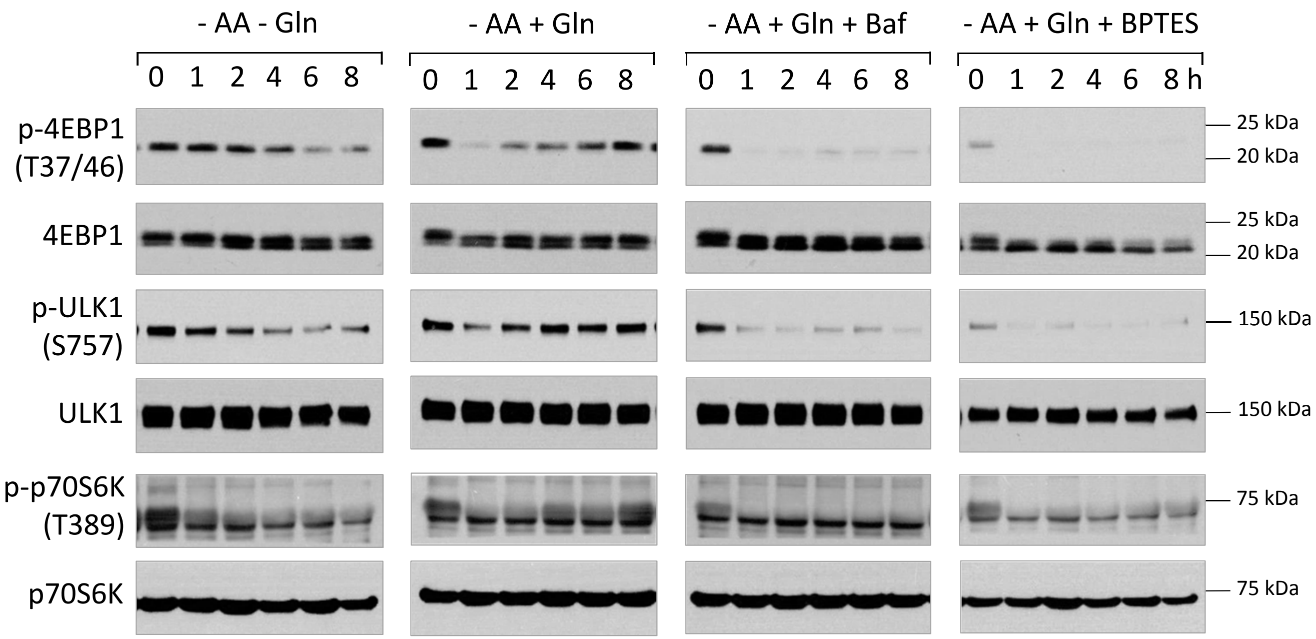

Supplementary Figure 1. Glutamine-mediated mTORC1 reactivation is abolished by inhibition of autophagy or glutaminolysis in DMEM
Wild type MEFs were starved of amino acids (in DMEM) with or without the supplementation of glutamine (4 mM) for the indicated durations in the presence or absence of bafilomycin (200 nM) or BPTES (10 µM). Changes in mTORC1 activity were assessed by immunoblotting of p-4EBP1, p-ULK1 and p-p70S6K.

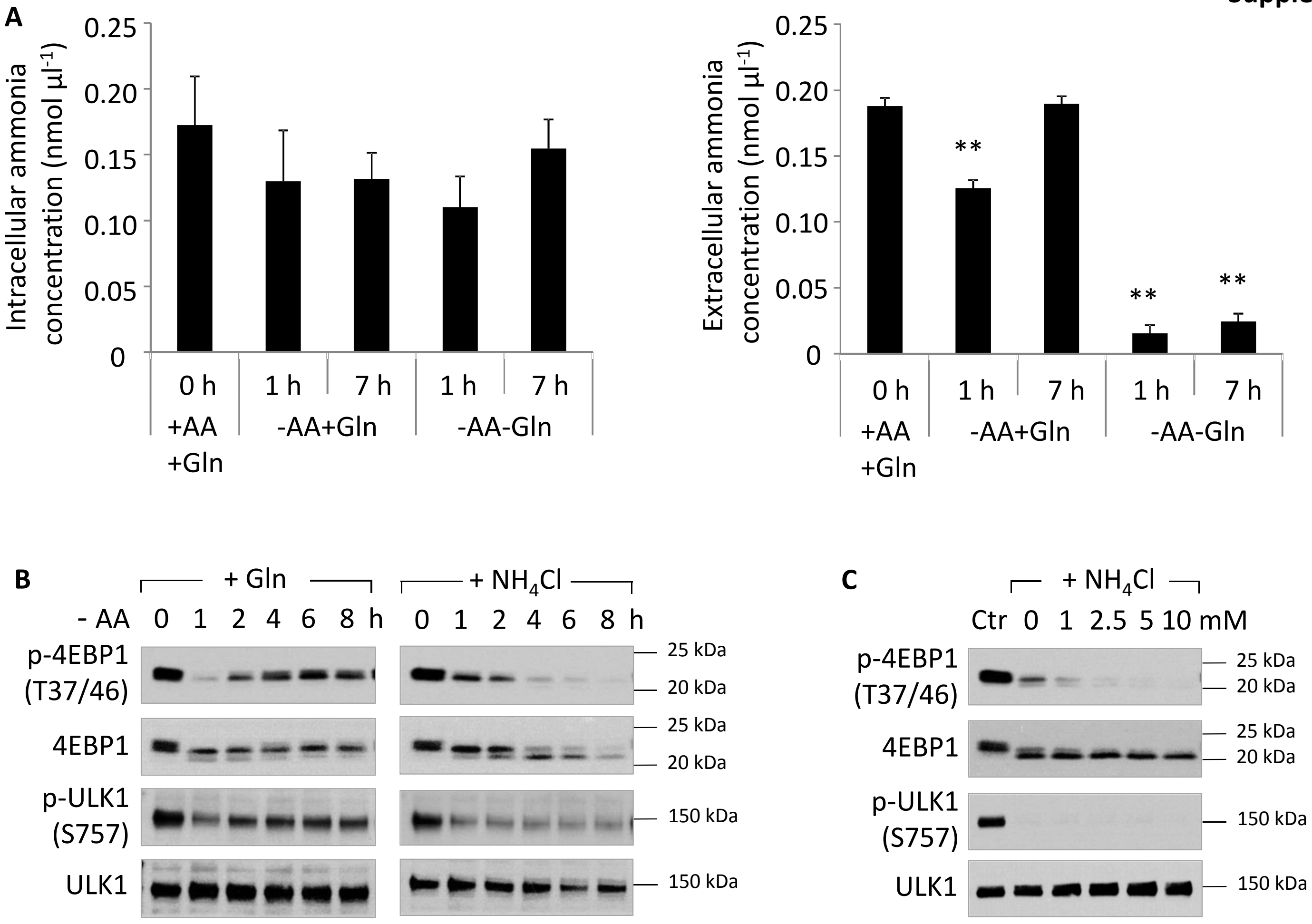

Supplementary Figure 2. Ammonia does not restore mTORC1 signaling during amino acid starvation
(A) Wild type MEFs were starved of amino acids with or without the supplementation of glutamine (4 mM) for the indicated durations, and intracellular and extracellular ammonia concentrations were determined. Data are the mean ± SEM of n = 4, **P ≤ 0.01 (in comparison to + AA + Gln at 0 h via Student's t-Test). (B) Wild type MEFs were starved of amino acids for the indicated durations in the absence or presence of ammonium chloride (2 mM). (C) Wild type MEFs were treated with amino acids and glutamine (control), or subjected to amino acid starvation without glutamine (for 4 h) followed by supplementation of ammonium chloride (for 4 h) at the indicated concentrations. Changes in mTORC1 activity were assessed by immunoblotting of p-4EBP1 and p-ULK1.

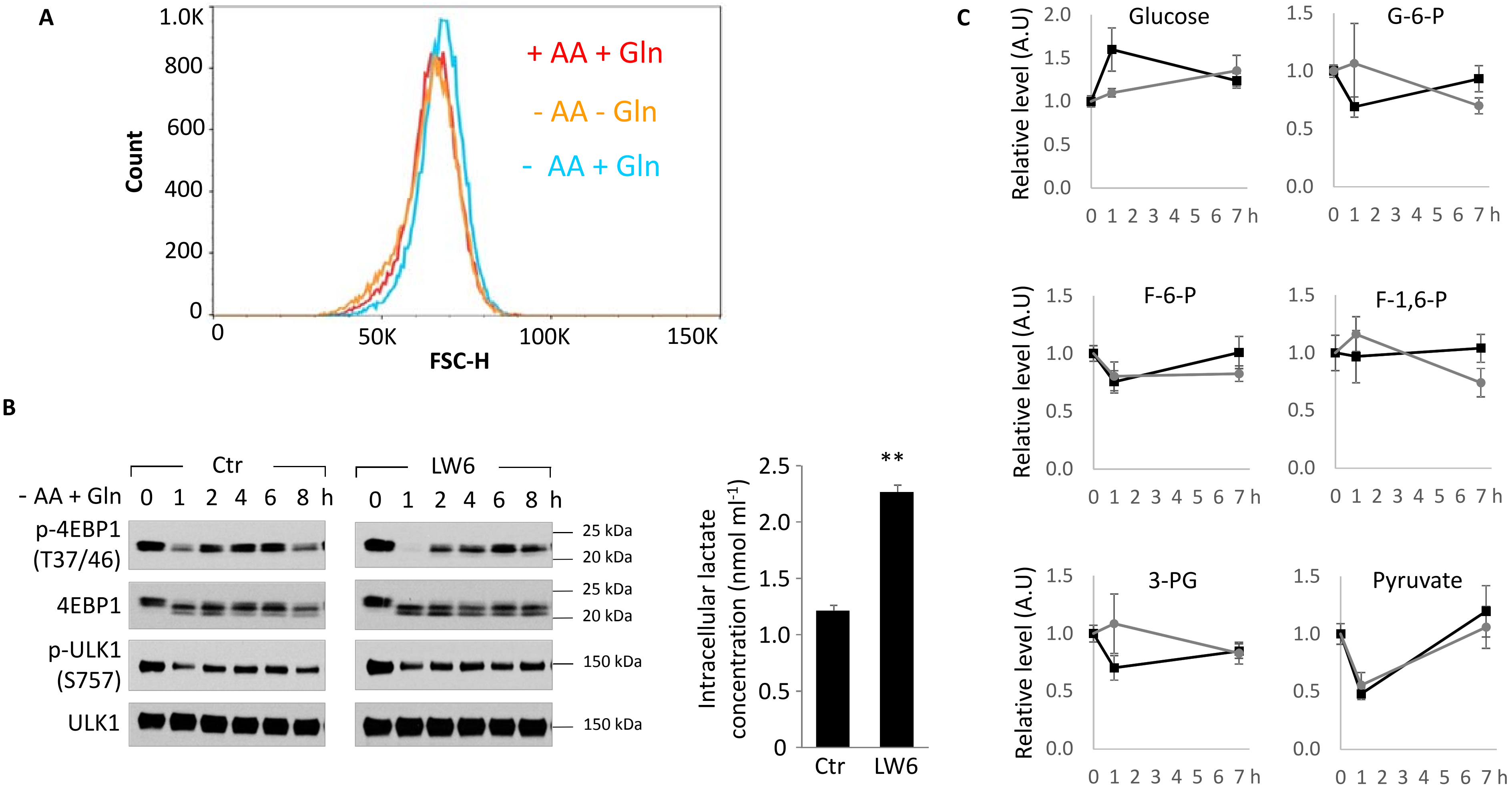

Supplementary Figure 3. Glutamine mediates mTORC1 reactivation independent of the malate-aspartate shuttle

(A) Wild type MEFs were harvested and subjected to flow cytometry analysis after 7 h incubation in the indicated conditions. Cell size was then evaluated by FSC-H. (B) Wild type MEFs were starved of amino acids with glutamine (4 mM) in the presence of LW6 (20 µM) for the indicated durations. Changes in mTORC1 activity were assessed by immunoblotting of p-4EBP1 and p-ULK1. Intracellular lactate concentration (after 7 h treatment) was determined by lactate assay kit. Data are the mean ± SEM of n = 6, **P ≤ 0.01 (in comparison to control group via Student's t-Test). (C) Wild type MEFs were starved of amino acids with (black line) or without (grey line) the supplementation of glutamine (4 mM) for the indicated durations. Individual glycolytic metabolite levels were measured from cell lysates by GC-TOF-MS. Data are expressed as the fold change of control cells (unstarved control, 0 h). Data are the mean ± SEM of n = 4 – 5.

Figure 1

Supplementary Figure 4

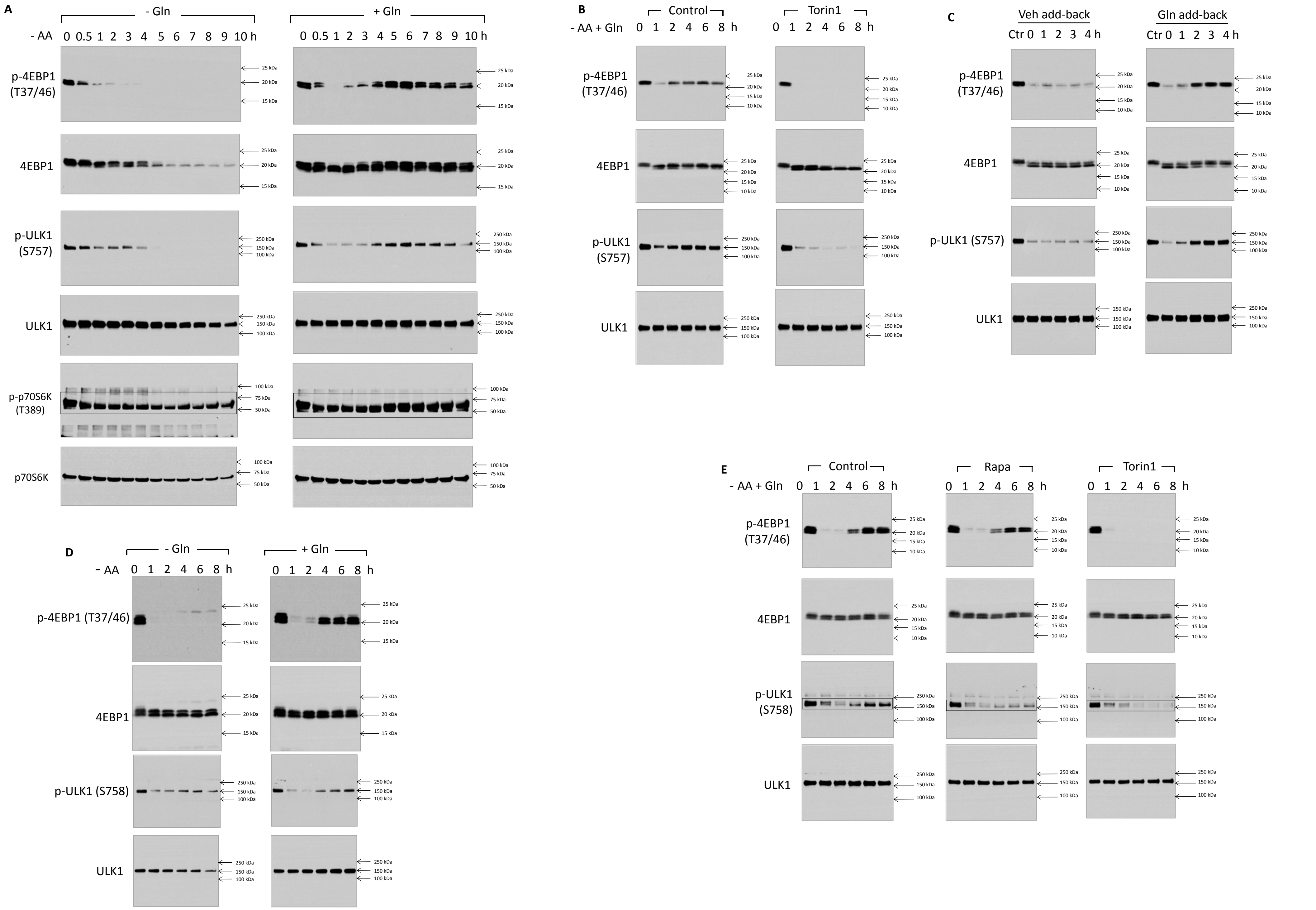

Supplementary Figure 4

Figure 2

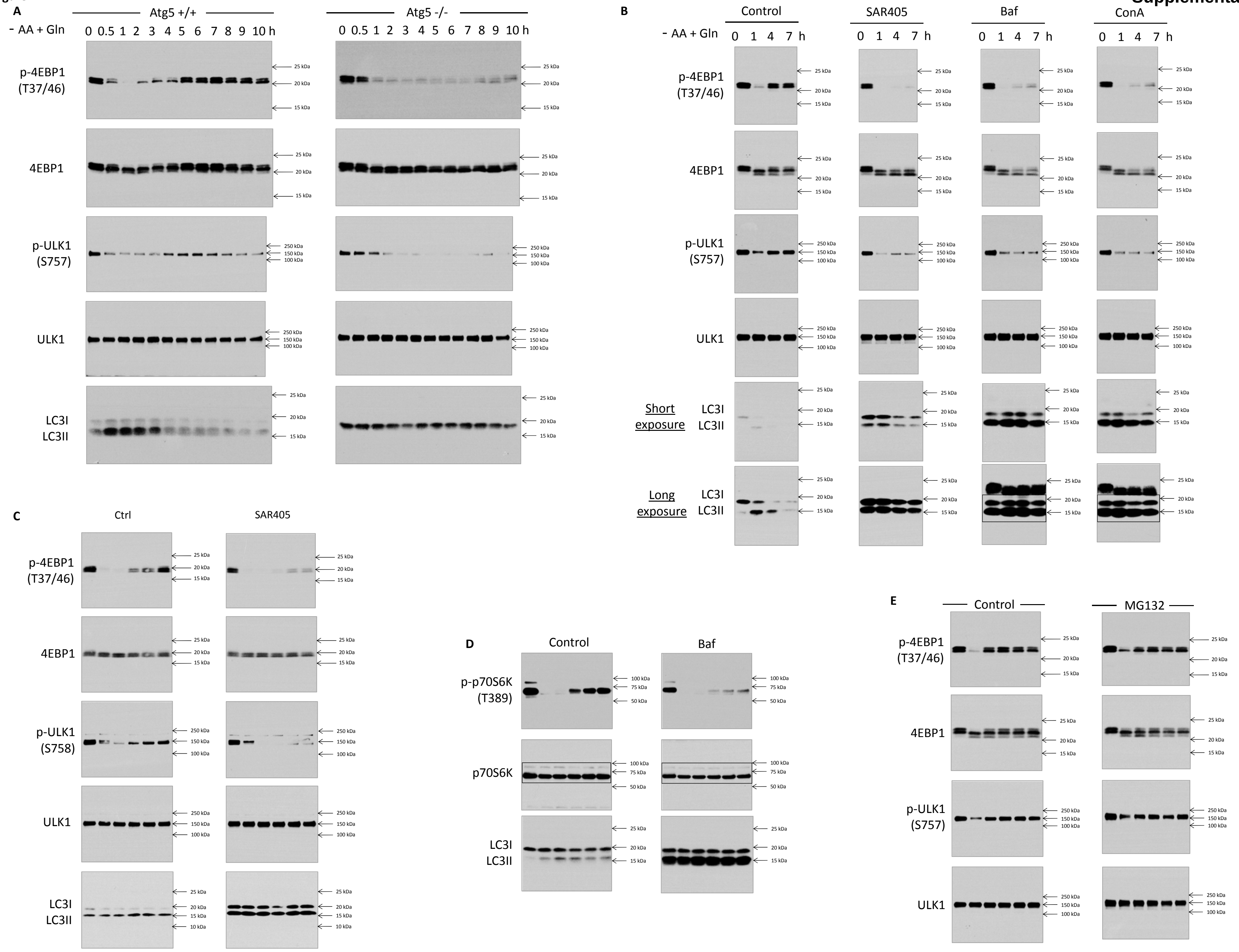

Supplementary Figure 4

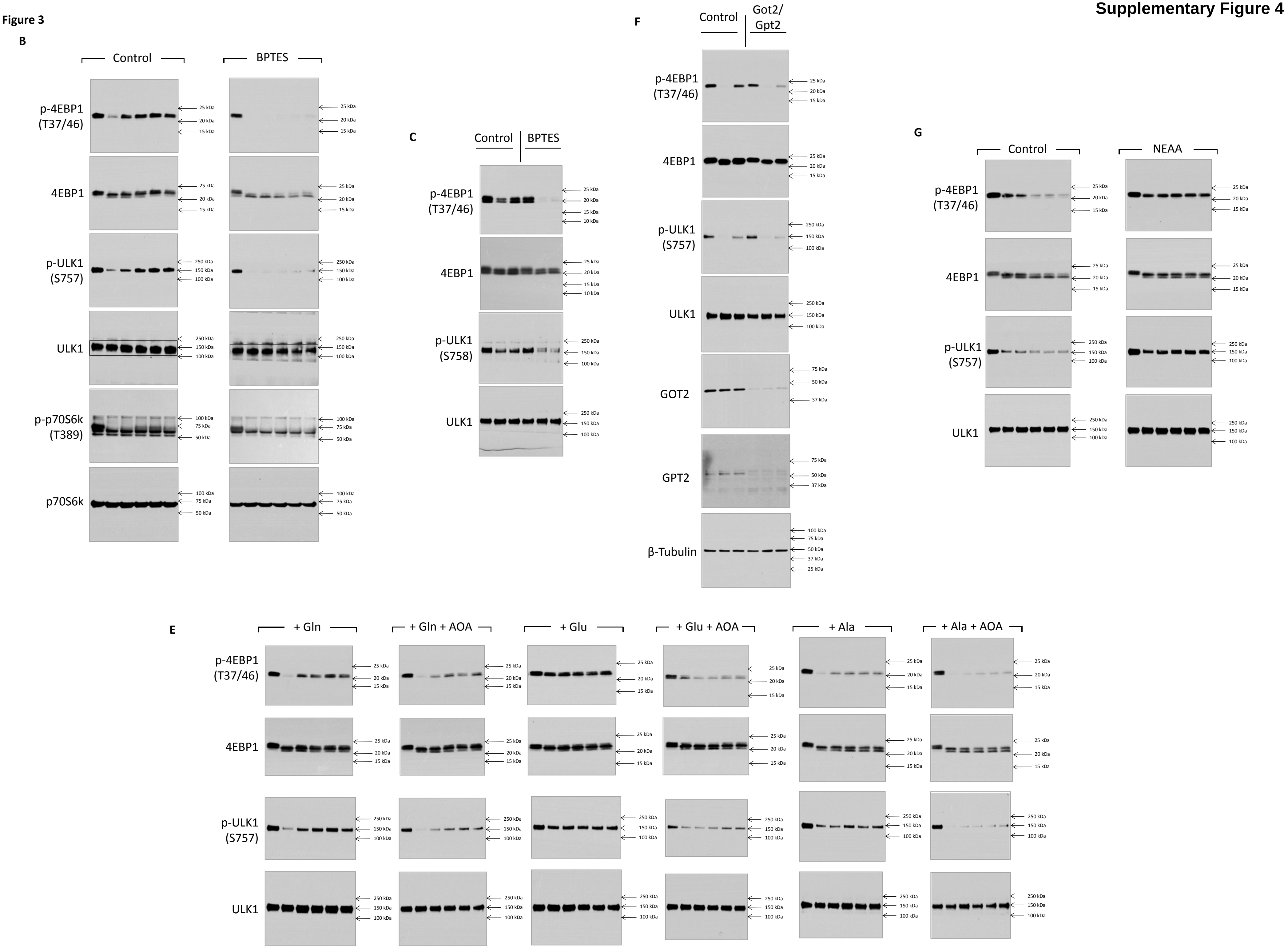

Supplementary Figure 4

Figure 4

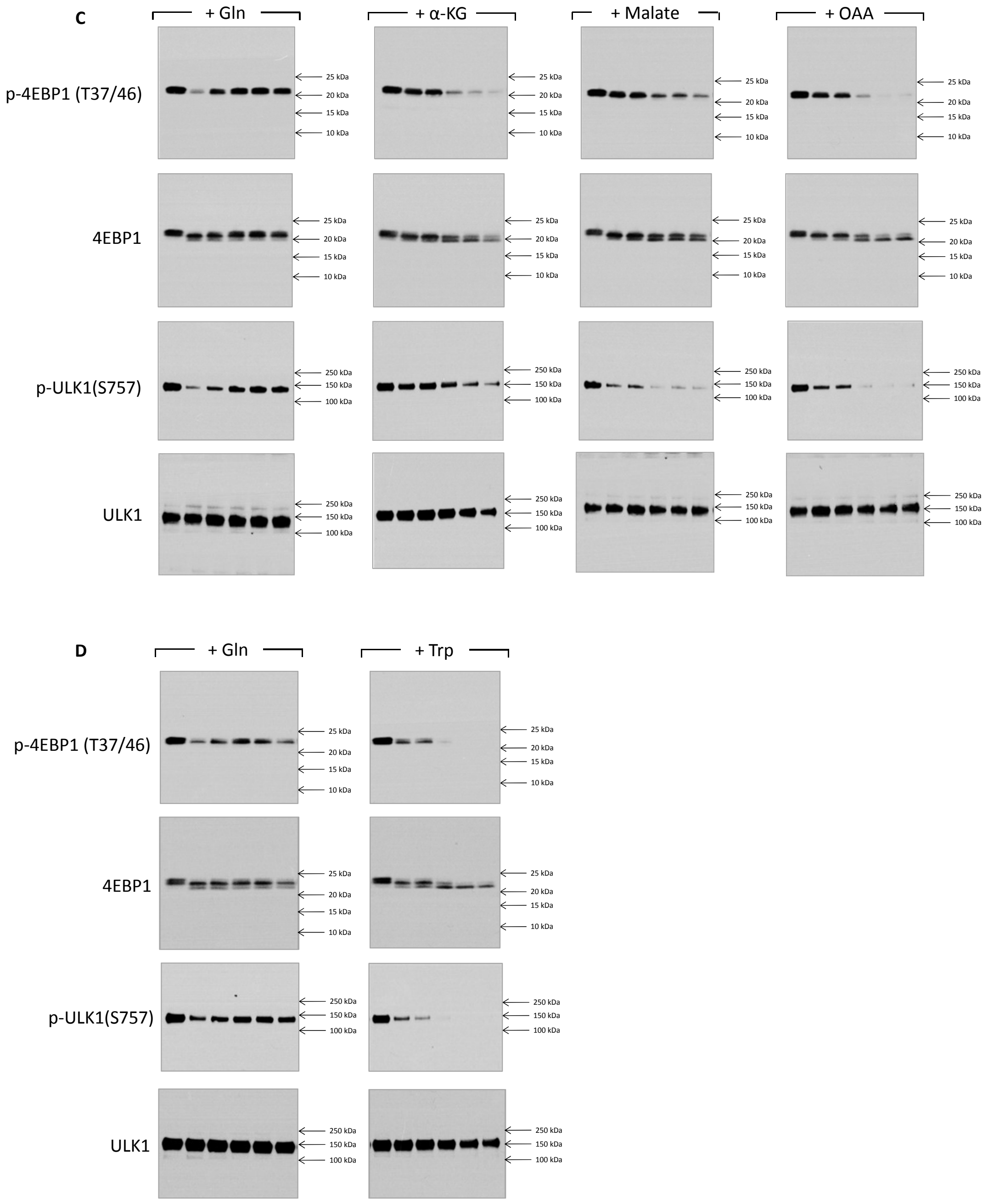

Figure 5

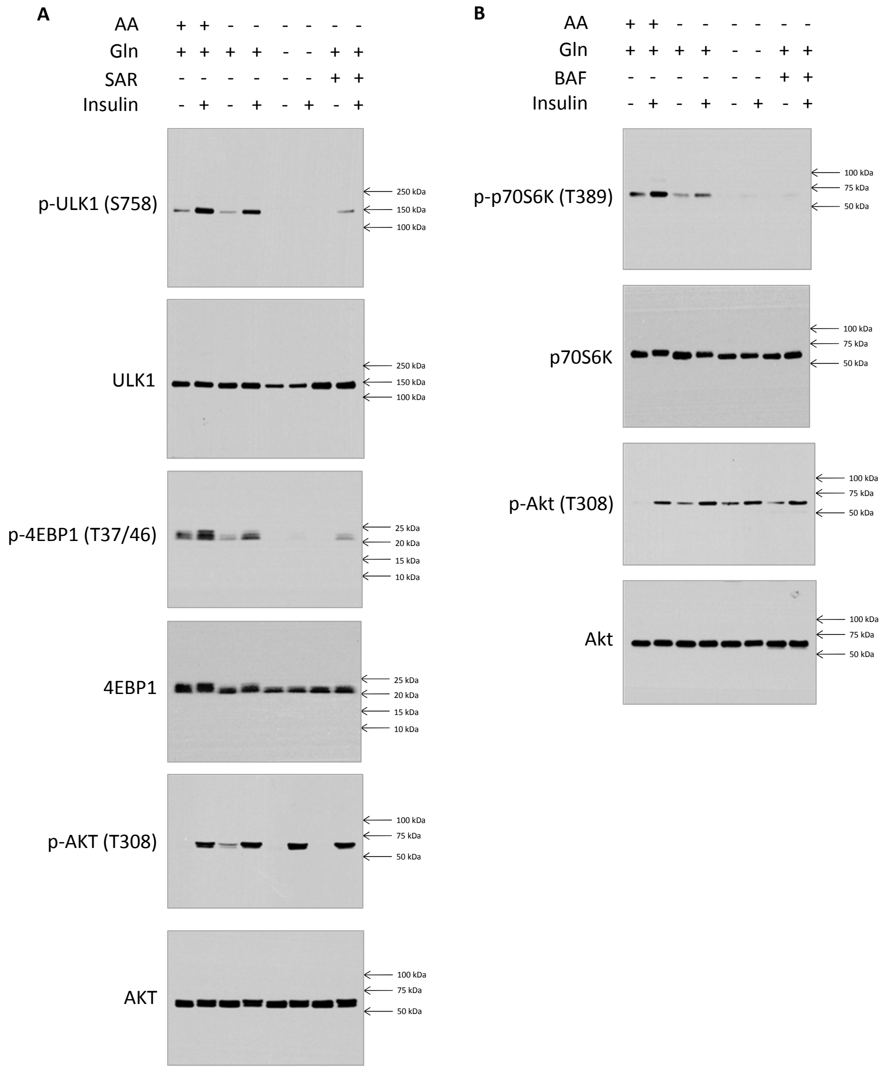

Supplementary Figure 1

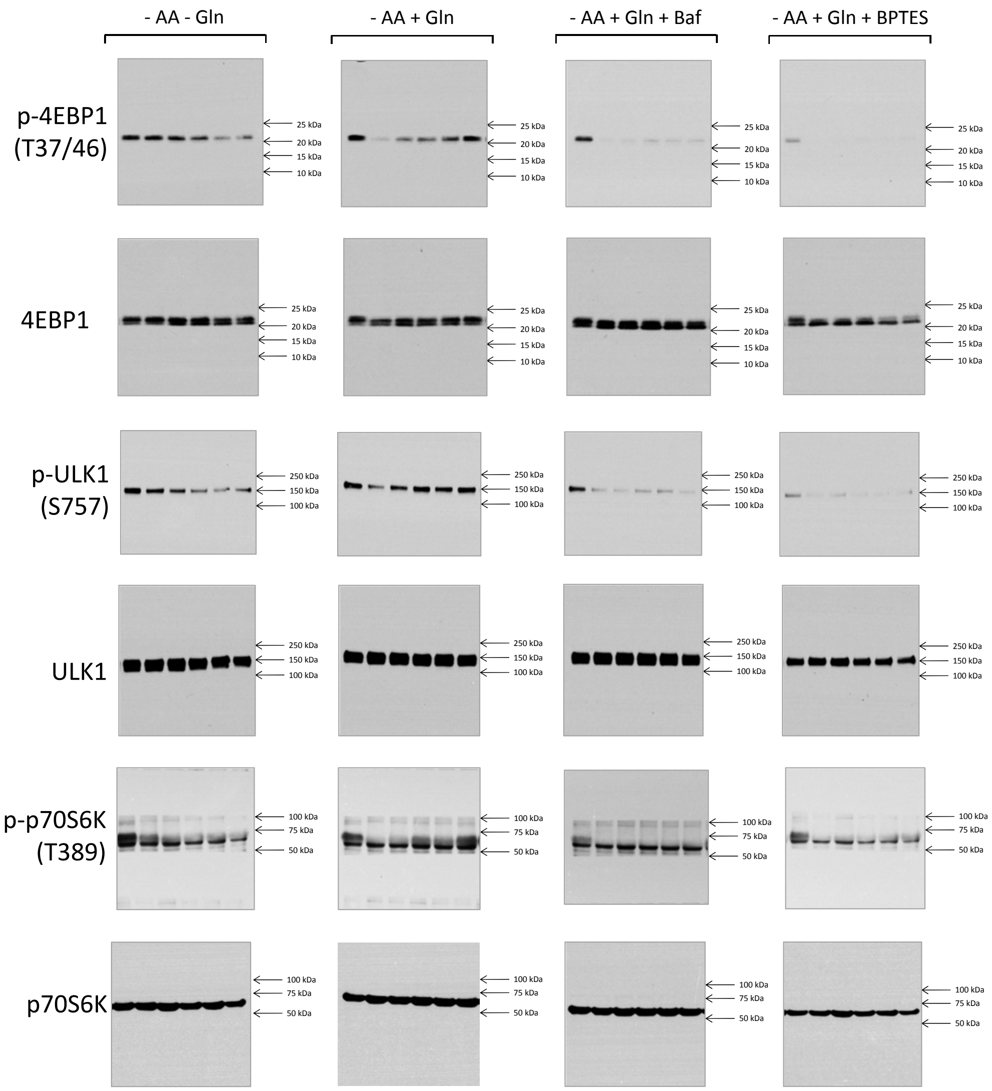

Supplementary Figure 2

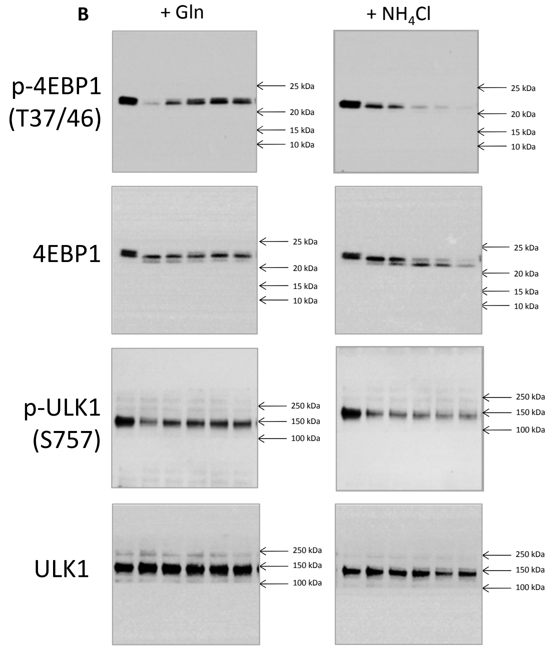

Supplementary Figure 4

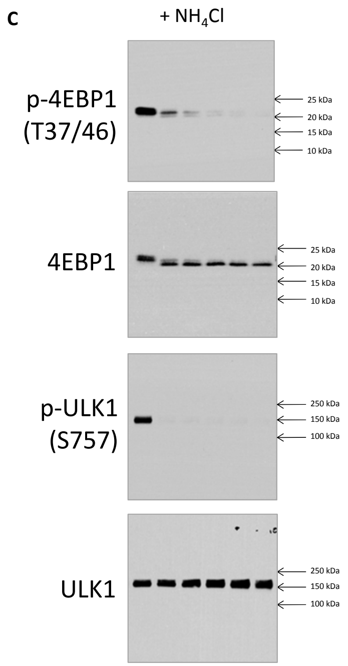

Supplementary Figure 3

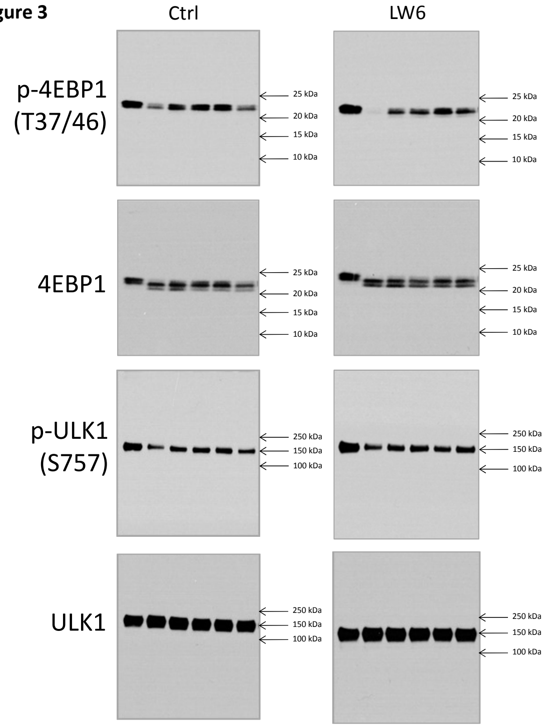

Supplementary Figure 4. Wider crop of immunoblots which are presented in the main paper.
